# Longitudinal Analysis of Adiponectin through 20-Year Type 1 Diabetes Duration

**DOI:** 10.1155/2015/730407

**Published:** 2015-04-09

**Authors:** Tamara J. LeCaire, Mari Palta

**Affiliations:** ^1^Department of Population Health Sciences, School of Medicine and Public Health, University of Wisconsin, Madison, WI 53726, USA; ^2^Department of Biostatistics and Medical Informatics, School of Medicine and Public Health, University of Wisconsin, Madison, WI 53726, USA

## Abstract

Little information exists on the trajectory and determinants of adiponectin, a possible insulin sensitizer and marker for inflammation and endothelial function, across the duration of type 1 diabetes. The Wisconsin Diabetes Registry Study followed an incident cohort ≤30 years of age when diagnosed with type 1 diabetes during 1987–1992 up to 20-year duration. Adiponectin was concurrently and retrospectively (from samples frozen at −80°C) measured for those participating in a 20-year exam (*n* = 304), during 2007–2011. Adiponectin levels were higher in females, declined through adolescence, and increased with age thereafter. Lower levels were associated with greater body weight and waist circumference and with higher insulin dose, especially at longer diabetes durations. Higher levels were associated with higher HbA_1_c and, at longer durations, with higher albumin-creatinine ratio. Adiponectin levels showed consistency within individuals that was not explained by these factors. We conclude that markers for insulin resistance are associated with lower adiponectin, and markers for potential microvascular complications are associated with higher adiponectin. The previously reported relationship with HbA_1_c remains largely unexplained. Additional individual specific factors likely also influence adiponectin level. The relationship between adiponectin and urinary protein excretion may enable identification of those predisposed to kidney disease earlier in type 1 diabetes.

## 1. Introduction

Adipose tissue, now considered an endocrine organ, produces inflammation and metabolism mediating cytokines [1]. One such adipokine, adiponectin, may be an endogenous insulin sensitize [2], necessary for regulation of insulin sensitivity and glucose homeostasis [3, 4]. Low levels have been consistently linked with obesity and predict the development of insulin resistance and type 2 diabetes [5, 6]. The role of adiponectin in type 1 diabetes is less clear. Some studies in children and most studies in adults have shown adiponectin to be higher in type 1 diabetes than in nondiabetic individuals and in those with type 2 diabetes [4, 7–11]. Nonetheless, relatively lower levels of adiponectin may also be related to insulin resistance in type 1 diabetes [10–15].

Adiponectin has been shown to have anti-inflammatory and antiatherogenic effects [3, 16] including enhanced nitric oxide production and vasodilation and reversal of the proinflammatory effects of tumor necrosis factor-alpha (TNF-*α*) on endothelial function [3]. It has been speculated that a compensatory mechanism may lead adiponectin levels to respond to inflammation and oxidative stress [8, 12, 14, 17]. Other factors that may affect adiponectin include peripheral hyperinsulinemia accompanying subcutaneous insulin administration or the chronic hyperglycemic state of type 1 diabetes [9, 10, 12]. A reduced clearance of adiponectin may contribute to higher levels found in individuals with advanced kidney disease [12, 14].

Most studies of adiponectin in type 1 diabetes have been cross-sectional and clinic based [4, 8, 18–20], and few included multicenter or population-based samples [9, 10, 12–14]. Limited longitudinal investigations have primarily followed younger individuals [7, 21, 22], and none have spanned durations from diagnosis during childhood through long-standing diabetes in adults. Consequently, there is little information on how adiponectin changes across longer type 1 diabetes duration and whether factors associated with adiponectin levels differ during early and later diabetes.

The Wisconsin Diabetes Registry Study (WDRS) is a population-based cohort of individuals followed up since diagnosis of type 1 diabetes through 20-year duration. Utilizing longitudinally stored plasma, we describe adiponectin levels across childhood through adulthood and investigate the relationship of adiponectin to markers of insulin resistance, glycemic control, correlates of inflammation, and endothelial function (microvascular changes in the kidney) as well as estimate consistency of levels within individuals. Our results reflect adiponectin determinants and tracking in individuals with a lower than expected level of complications due to contemporary diabetes care [23, 24] and before advanced vascular damage for nearly all.

## 2. Methods

### 2.1. The WDRS Population

Residents of a geographically defined region of central and southern Wisconsin ≤30 years of age with newly diagnosed type 1 diabetes during 1987–1992 were eligible for enrollment in WDRS. Type 1 diabetes was defined by classic symptomatology with exogenous insulin administration. Subjects were referred by provider, family member, or themselves, as described previously [24]. Five hundred eighty-nine subjects (81%) with continued insulin use were enrolled and followed up.

Over the next 20 years, the WDRS cohort was comprehensively followed by telephone and mail questionnaires for diabetes management and health history, mailed or in-person blood kits for glycemic control, and in-person examinations for anthropometric measures and outcomes [23–25]. For 304 individuals participating in a 20-year exam during November 2007 through July 2011, stored plasma samples from the current exam as well as samples collected previously at 1-, 4-, 7-, or 9-year exams were tested for adiponectin. Samples from 1 to 5 (mean 3.43) exams yielded 1043 samples tested.

### 2.2. Data Collection

Standard protocols were followed across all exams. Height and weight (without shoes) were measured by a standard stadiometer height rod fixed to a Healthometer physician beam scale (Health O Meter, Inc., Bridgeview, IL). Waist and hip circumferences (cm) were measured in duplicate and averaged. BMI (kg/m^2^) and waist-hip ratio (WHR) were calculated. Two seated blood pressure measurements in the right arm were obtained (and averaged) with appropriate cuff selection using a Random Zero sphygmomanometer (Hawksley and Sons, Sussex, UK), five minutes after cuff placement and again after a five-minute rest [26].

Questionnaires provided information on diabetes self-management and medication use. Through the 9-year exams, adolescents (girls aged 10–16 years and boys aged 10–18 years) identified their Tanner stage of pubertal development [27]. Blood specimens by venipuncture and a 24-hour (baseline and 4-year exams) or timed overnight (7-, 9-, and 20-year exams) urine specimen were requested and stored at −80°C.

#### 2.2.1. Laboratory Assays

At 20 years, assays were performed by Fairview Diagnostic Laboratories at the University of Minnesota (UMN) (Minneapolis, MN). Total adiponectin (in mg/L) was tested using the Quantikine Human Adiponectin/Acrp30 (ELISA) Immunoassay (R&D Systems, Minneapolis, MN). The interassay CV was 5.8–6.9% and the intra-assay CV was 2.5–4.7%.

Blood samples at 20 years were analyzed within 7 days of collection for Diabetes Control and Complications Trial- (DCCT-) equivalent glycosylated hemoglobin A1c (HbA_1_c, %) by automated high performance liquid chromatography at the UMN. The interassay CV was 1.7% at normal HbA_1_c levels (5.4% or 36 mmol/mol) and 1.0% at elevated (10.8% or 95 mmol/mol) HbA_1_c levels. The intra-assay CV was 1.5% at 4.7% HbA_1_c (28 mmol/mol) and 0.4% at 10.1% (87 mmol/mol) HbA_1_c. Previous exam blood samples were tested for total glycosylated hemoglobin (GHb, %) by the study's central laboratory in duplicate and repeated when the duplicate CV was >5% (by GlycAffin microcolumn affinity chromatography, Isolab, Akron, OH) [25]. From a validation study with split samples, an equation was developed to convert GHb to DCCT-equivalent HbA_1_c [23].

Urine albumin was determined by double-antibody ^125^Iodine radioimmunoassay (Diagnostic Products Corporation, Los Angeles, CA) for 1- to 9-year exam samples and by nephelometry using a Behring ProSpec analyzer (Dade Behring, Marburg, Germany) for 20-year exam samples. Urine creatinine was measured in a Beckman Creatinine II analyzer (Beckman Instruments, Fullerton, CA) using the picrate acid color reaction and the Jaffe rate technique through 9 years and by the Roche enzymatic method (Roche Diagnostics Corporation, Indianapolis, IN) on a Roche Modular P Chemistry Analyzer at 20 years. Urine albumin to creatinine ratios (UACR) (in mg albumin/g of creatinine) were determined. Inter- and intra-assay coefficients of variation were <6% for urine albumin and <5% for urine creatinine at all exams.

### 2.3. Statistical Methods

The cohort was described by means, standard deviations, medians, and percentages using SAS version 9.2 [28]. Log transformations (log⁡_*e*_⁡(*x* + 1)) of adiponectin, UACR, and insulin dose were used to normalize distributions.

Adiponectin was plotted on age at exam and years of duration by age at diagnosis groups, with smoothing by polynomial splines (SAS proc gplot). The relationship of adiponectin to age and sex was also modeled by a repeated measures model fitted by SAS proc mixed with a compound symmetry variance structure and robust standard errors [29]. Relationships between adiponectin and potential determinants across all ages were screened by Spearman correlations. Factors found significantly correlated with adiponectin were added to a model including age and sex stepwise in order of strength of their correlation, retained in the model if significantly related at *P* ≤ 0.05, and removed if becoming nonsignificant (*P* > 0.05). Two-way interaction effects, including those of diabetes duration with each variable, were considered between all significant factors and also retained at *P* ≤ 0.05. Models omitting and adding variables were explored to compare results with findings in the literature. Results are presented as regression coefficients and as the percent difference in adiponectin associated with meaningful number of units difference in predictors. Consistency of adiponectin levels over time within individual (tracking) was assessed by the within-individual correlation estimated from the final compound symmetry model. In a sensitivity analysis, results of modeling were compared with those of a model fitted to individuals who had at least 2 data points including at least 1 in the first 4 years.

## 3. Results

The majority (96%) of individuals in the WDRS cohort with adiponectin measures for the current analysis (*n* = 304) had 2 or more observations. They were on average 30.9 years of age at the 20-year exam and 11.3 years of age at diagnosis. Most (97%) were white and half were male. At each exam, subjects with adiponectin tested were representative of those examined and had baseline characteristics similar to all subjects.

Characteristics of the cohort across exams are presented in Table 1. Weight and waist circumference increased with age. HbA_1_c and insulin dose were greatest at the 4–9-year exams where many individuals were passing through pubertal stages. Intensive insulin management (insulin pump or 3 or more injections/day) was increasingly practiced and reached 94% by the 20-year exam. Antihypertensive and lipid lowering medications became more prevalent by 9 years and reached 29% and 23%, respectively, by 20 years. Microalbuminuria (UACR >30 but <300 mg/g) was found in 3–8% and macroalbuminuria (UACR >300 mg/g) was observed at the 9- and 20-year exams only (1.7% and 4.3%, resp.).

**Figure 1:**
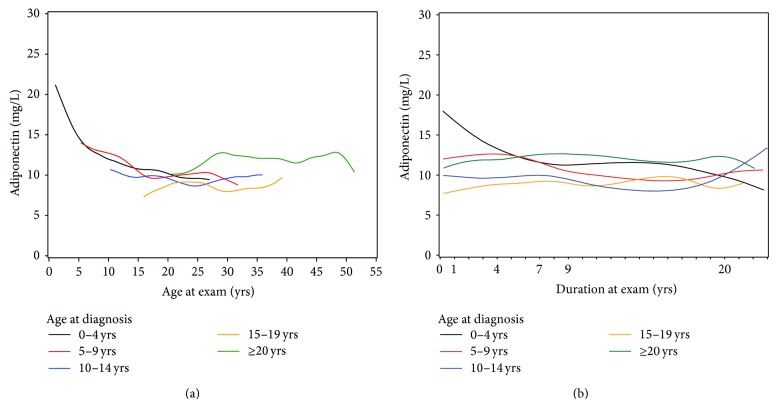
Adiponectin for age at diagnosis groups by (a) age and (b) diabetes duration at exam. Age at diagnosis groups 0–4, 5–9, 10–14, 15–19, and ≥20 years of age.

Mean and median adiponectin were 11.9 and 10.2 mg/L at the 1-year exam and 10.2 and 8.5 mg/L at the 20-year exam, respectively. Plotting mean adiponectin on age at exam (Figure 1(a)) and duration (Figure 1(b)) indicated a decline with age and through 9-year duration for those 0–15 years of age at diagnosis. A repeated measures regression model confirmed decline in log adiponectin up to age 20 (of 3.3% per year, *P* < 0.0001). Adiponectin was more stable after this age. Females had higher adiponectin levels than men with a difference of 16% at age 20.

In order, waist, weight, BMI, systolic blood pressure, Tanner stage, lipid medication use, diastolic blood pressure, log UACR, HbA_1_c, antihypertensive medication use, intensive insulin management, insulin pump use, and log insulin dose were found significantly correlated with adiponectin. WHR was collinear with waist and weight, and the latter two showed stronger associations with adiponectin.

Due to some missing data (primarily for UACR), 892 observations were included in the final model for 300 subjects, 87% of whom contributed 2 or more measurements. Results of the final model are shown in Table 2. Weight and waist circumference were negatively associated with adiponectin, accounting for 3.5% (95% CI 2.3–5.2%) and 3.7% (95% CI 1.1–5.8%) lower levels per 5 kg or cm increase, respectively. Adjusting for body habitus reversed the previously negative relationship of adiponectin to age during childhood and adolescence. The resulting increase in adiponectin with age was stronger in females than males (*P* = 0.004 for the interaction) and reflects the high adiponectin level of individuals diagnosed after age 20, as seen in Figure 1(a). Tanner stage 2 was associated with higher adiponectin levels than pre- or postpubertal stages (*β* = 0.126, *P* = 0.01). Adiponectin levels were greater by 4.1% (95% CI 2.4–5.6%) for a 1% higher HbA_1_c (*P* < 0.0001). Several predictors had opposite relationships with adiponectin at early and later diabetes durations. Log UACR was negatively related with log adiponectin at 1–4-year duration but a positive relationship emerged after 7 years (*P* < 0.0001 for interaction). At 20-year duration, adiponectin was higher by 8.5% (95% CI 5.4–11.7%) per 1% higher UACR. Log insulin dose was positively related with log adiponectin during early diabetes, but at 9 and especially 20 years a negative relationship emerged (*P* = 0.030 for interaction). The final compound symmetry model showed significant residual within-individual tracking (*r* = 0.59) of adiponectin over time.

Further investigation indicated that as high HbA_1_c was associated with higher adiponectin and coincided with pubertal change in body habitus, removing HbA_1_c from the model introduced the appearance of a body weight by age interaction. Furthermore, as HbA_1_c was positively correlated with insulin dose adjusting for HbA_1_c was necessary to identify the negative association of adiponectin with higher insulin dose at older age.

Limiting analyses to those with repeated measures starting at 1–4-year duration showed results very similar to those presented from the final model, with only a slightly stronger effect of waist circumference and pubertal stage.

## 4. Discussion

Our long follow-up and large sample size allowed us to assess multiple determinants of adiponectin across a long duration of type 1 diabetes. Besides age and gender we found body composition, insulin dose, urinary protein, and glycemic control related to adiponectin levels in type 1 diabetes, some with persistent association across age and duration and others stronger at longer duration. Higher insulin dose was especially strongly related to lower adiponectin and microalbuminuria to higher adiponectin at 20-year duration. Our results support pathways related to insulin resistance as well as to inflammatory response and endothelial function. As in other reports, we also find higher adiponectin with worse glycemia [9, 10]. This has been suggested to be related to feedback loops tied to downstream inflammatory or oxidative stress effects of the chronic hyperglycemic state. We found that, due to the interrelationship of glycemia with insulin resistance and adolescence, it was necessary to jointly consider these factors to correctly discern their associations with adiponectin in type 1 diabetes.

In addition, our longitudinal data allowed us to evaluate the consistency of adiponectin levels in an individual over time. After removing the influence of measured time varying and individual specific factors, adiponectin showed substantial tracking from childhood through adulthood, shortly after type 1 diabetes diagnosis up to 20-year duration. Similar tracking was found over the much shorter period of 5 years in a previous study [7]. The finding points to further individual level characteristics, either unmeasured or imprecisely captured, influencing adiponectin levels. A genetic component even in the complicated milieu of type 1 diabetes [7, 14] has been suggested.

Consistent with lower adiponectin being a marker of higher insulin resistance and consistent with previous studies [10, 12, 14], we found negative associations between adiponectin and weight and waist circumference. We also found adiponectin levels to be higher during Tanner stage 2. These relationships explained a decline in adiponectin during childhood and across adolescence as weight and waist circumference increased and puberty progressed. In nondiabetic children, a progressive decline in adiponectin in childhood during the prepubertal years has been well documented [30]. Previous results for trends in children and adolescents followed up beyond the first year of type 1 diabetes have been mixed, perhaps due to smaller sample size in some studies [7, 21]. Galler et al. [7] suggested that regulation of adiponectin could depend on pubertal status at type 1 diabetes onset. Our data support this as individuals diagnosed after age 20 had higher adiponectin levels.

Most studies of nondiabetic children report no difference in adiponectin by gender up to 9 years of age. A decline during puberty in males, attributed to increasing androgen levels, has been suggested as the point at which levels begin to diverge by gender [30]. We report a difference by gender at all ages but one that widened with age, leading to a quite strong gender difference by age 20. Consistent with this, studies of adults with and without type 1 diabetes show women to have higher adiponectin levels than men [10, 31, 32].

Based on previous work, it has been speculated that adiponectin may be less important to the development of insulin sensitivity in children [7, 8, 18, 22]. However, when glycemic level was taken into account, the relationship of body composition with adiponectin did not differ by age or duration. On the other hand, a strong negative relationship between adiponectin and log insulin dose did increase with duration, even more strongly so after adjusting for all other factors, including body composition. This suggests that adiponectin may be more strongly related to insulin resistance at longer duration of type 1 diabetes.

It has been speculated that adiponectin could be “pathogenically related to the development of microvascular complications” [14, page 1916]. In particular, adiponectin has been found positively associated with prevalent kidney disease or progression to more advanced stages in individuals with and without type 1 diabetes [12, 14, 33]. We found an association between urine albumin level and adiponectin that varied by duration, with a positive relationship between higher protein excretion and adiponectin only becoming evident after 7 or more years of duration. However, our finding of higher adiponectin with worse and worsening kidney function is also very consistent with rising adiponectin being a compensatory mechanism in response to increased inflammation and oxidative stress [8, 14, 16]. As such, adiponectin may serve as an early marker for microvascular complications.

We are the first to show higher HbA_1_c persistently related to higher adiponectin shortly after diagnosis through 20-year diabetes. A positive relationship between HbA_1_c and adiponectin in type 1 diabetes has been noted previously, primarily in children or those without advanced complications [10, 18, 21], but not in all investigations [4, 7, 9, 14, 19, 20]. Reports with negative findings may have been limited by cross-sectional study design with one HbA_1_c measurement. Further, a relationship between adiponectin and HbA_1_c could have been masked by a strong relationship between nephropathy and adiponectin at later durations in a cross-sectional investigation [14].

Adiponectin may respond to hyperglycemia resulting from glucose production by the liver by feedback loop response [4, 10]. Specifically, adiponectin may work to sensitize the liver to insulin to prevent glucose production [34]. Hepatic insulin resistance appears to be a key component of insulin resistance in type 1 diabetes [35], and further research on the impact of liver metabolism and hepatic insulin resistance on adiponectin levels in type 1 diabetes may be warranted.

## 5. Strengths and Limitations

The Wisconsin Diabetes Registry Study provided a unique opportunity to evaluate adiponectin levels longitudinally in individuals with type 1 diabetes up to 20-year diabetes duration. Few population-based studies have followed individuals from diagnosis for this period of time. Yet, there are limitations to our analysis. Not all individuals contributed the full set of longitudinal observations. Nonetheless, sensitivity analyses of a subset with longest follow-up showed very similar results to those from the final model. Our sample size, while larger than most of the few previous longitudinal investigations, may have also limited our ability to investigate the impact of insulin management on adiponectin simultaneously with glycemic control. Urinary albumin-creatinine ratio at each exam was determined from one sample, and assay methods changed over time and there was some missing data. Consequently, our results may underestimate the relationship between adiponectin and UACR.

Although adiponectin has previously been found stable after several years of deep-freezing [20, 36], samples in the current analysis had been stored for longer periods, up to 20 years after diabetes onset, and possible changes cannot be ruled out. Adiponectin has been shown to have “relatively low biovariability” with no regular diurnal changes and no significant change with fasting versus postprandial status [36]. The variance of adiponectin in replicate sampling as estimated from our data was 4–4.8 (SD 2–2.2) indicating that part of the residual model variance is due to measurement error. Hence, tracking and the variability in adiponectin explained by the predictors in our model were likely somewhat underestimated.

Finally, we did not consider adiponectin subtypes. Adiponectin circulates in three different subforms, and the high-molecular-weight (HMW) subform is believed to be the primary biologically active form. Previous research has found that the elevation in adiponectin with type 1 diabetes is mainly explained by an elevation in the HMW subform [20]. However, the elevation was unaffected by gender and diabetic kidney disease. Further research is needed to examine other determinants of the HMW subform.

## 6. Summary

In conclusion, a decline in adiponectin in children and adolescents with type 1 diabetes was strongly related to increasing weight and waist circumference with aging and puberty progression. Adiponectin may be more strongly related to insulin sensitivity in adults, as previously suggested [18]. In support of this, correlates of insulin resistance, insulin dose, and waist circumference were strongly related to adiponectin levels in adults at 20 years. While a relationship with insulin resistance emerged, it is partly obscured by a positive relationship between adiponectin and glycemia. In general the determinants of adiponectin in type 1 diabetes appear complex. Evidence of tracking of adiponectin within individuals was noted, and further investigation to determine other individual characteristics influencing adiponectin could be important. The association between adiponectin and urinary protein suggests it may be possible to identify those predisposed to develop kidney disease earlier in type 1 diabetes.

## Figures and Tables

**Table 1:** Characteristics for Wisconsin Diabetes Registry Study participants with adiponectin tested.

Characteristics	1-year exam	4-year exam	7-year exam	9-year exam	20-year exam
*N*	184	231	137	187	304
Male, %	52%	49%	48%	53%	49%
White, %	98%	98%	97%	98%	97%
Diabetes duration, years	0.4 (0.2)	3.4 (0.3)	6.5 (0.3)	9.3 (0.7)	19.7 (1.2)
Age at diagnosis, years	10.9 (6.8)	11.2 (6.8)	10.8 (6.5)	11.4 (7.3)	11.3 (7.0)
0–4 years	18%	16%	18%	18%	17%
5–9 years	33%	32%	31%	32%	32%
10–14 years	25%	28%	27%	23%	25%
15–19 years	12%	12%	13%	14%	12%
≥20 years	11%	11%	10%	13%	13%
Age at exam, years	11.3 (6.8)	14.6 (6.8)	17.3 (6.5)	20.8 (7.4)	30.9 (7.0)
Tanner					
1	58%	33%	16%	5%	0%
2	2%	5%	10%	4%	0%
3	7%	7%	5%	7%	0%
4	13%	20%	16%	13%	0%
5	19%	34%	53%	71%	100%
Intensive insulin management, %	15%	46%	67%	74%	94%
Insulin pump, %	0%	0%	0.7%	11%	48%
Insulin dose (units/kg/day)	0.49 (0.30)	0.77 (0.27)	0.85 (0.26)	0.88 (0.29)	0.75 (0.30)
HbA_1_c, %^*^	7.3 (1.7)	9.0 (1.8)	9.1 (1.6)	8.8 (1.7)	8.0 (1.5)
HbA_1_c, mmol/mol^*^	56 (18.6)	75 (19.7)	76 (17.5)	73 (18.6)	64 (16.4)
BMI, kg/m^2^	19.5 (3.5)	21.3 (4.2)	22.8 (4.3)	25.2 (5.0)	28.3 (5.9)
Weight, kg	42 (22)	53 (22)	61 (20)	72 (20)	84 (20)
Waist, cm	65 (11)	71 (11)	76 (11)	83 (12)	86 (14)
Waist-hip ratio	0.84 (0.06)	0.81 (0.06)	0.81 (0.05)	0.81 (0.06)	0.82 (0.08)
Systolic/diastolic BP, mmHg	101 (13)/64 (10)	105 (13)/67 (9)	108 (12)/73 (8)	112 (11)/71 (9)	122 (13)/77 (9)
Micro/macroalbuminuria, %^*^	3.4%/0%	7.5%/0%	4.1%/0%	4.0%/1.7%	6.8%/4.3%
log_*e*_⁡ (UACR), units^*^	2.1 (0.6)	2.1 (0.8)	2.0 (0.7)	2.2 (1.0)	2.1 (1.3)
Antihypertension med. use, %	0%	0.4%	1%	7%	29%
Lipid lowering med. use, %	0.5%	0.4%	0%	3%	23%
Adiponectin, mg/L	11.9 (6.4)	11.4 (5.5)	11.3 (5.5)	10.2 (5.7)	10.2 (7.1)
Median adiponectin, mg/L	10.2	10.4	9.8	9.0	8.6

Entries are means (SD) and %. ^*^Missing data; *n* with data: for HbA_1_c, *n* = 159 at 1 yr; for UACR, *n* = 148 at 1 yr, *n* = 201 at 4 yr, *n* = 122 at 7 yr, *n* = 175 at 9 yr, and *n* = 278 at 20 yr exam.

**Table 2:** Results from the longitudinal model predicting log adiponectin.

Predictor	Estimate	*P* value	Percent difference in adiponectin	95% CI for percent difference
Age, per 1 year				
For females	**0.019**	0.002^*^	**1.9**	**1.1, 2.7**
For males	**0.010**		**1.0**	**0.3, 1.7**
Female versus male				
At 10 years of age	0.047	0.002^*^		
At 20 years	**0.137**		**14.7**	**5.6, 24.5**
At 30 years	**0.226**		**25.3**	**13.0, 39.1**
Tanner stage of 2	**0.112**	0.024	**11.8**	**0.8, 24.1**
Weight, per 5 kg	**−0.038**	0.004	**3.7**	**2.3, 5.2**
Waist, per 5 cm	**−0.036**	0.021	**3.5**	**1.1, 5.8**
HbA_1_c, per 1%	**0.040**	<0.0001	**4.1**	**2.4, 5.6**
HbA_1_c, per 10 mmol/mol	**0.036**	<0.0001	**3.7**	**2.1, 4.7**
log_*e*_⁡ (insulin dose), per 1 unit		0.030^*^		
At 1-year duration	0.169			
At 4 years	0.093			
At 7 years	0.016			
At 9 years	−0.034			
At 20 years	−0.314			
log_*e*_⁡ (UACR), per 1 unit		<0.0001^*^		
At 1-year duration	−0.026			
At 4 years	−0.009			
At 7 years	0.008			
At 9 years	0.019			
At 20 years	**0.082**		**8.5**	**5.4, 11.7**

Results from repeated measures model with compound symmetry variance matrix and robust standard errors.

^*^ *P* value for interaction terms. **Bold** indicates significance at *P* < 0.05.
